# Psychological and social consequences of deafblindness for siblings: a systematic literature review

**DOI:** 10.3389/fpsyg.2024.1102206

**Published:** 2024-04-25

**Authors:** Marine Arcous, Rémy Potier, Nathalie Dumet

**Affiliations:** ^1^Centre de Recherche Psychanalyse, Médecine et Société, Université Paris Cité, Paris, France; ^2^Centre de Recherche en Psychopathologie et Psychologie Clinique, Université Lumière Lyon 2, Lyon, France

**Keywords:** deafblindness, siblings, family, psychological consequences, social experiences, siblings’ psychotherapeutic interventions

## Abstract

The onset of deafblindness profoundly impacts both the individual with this condition and the individual’s family, including siblings. While current studies have primarily focused on the impact felt by parents or spouses, the distinct experiences of siblings have received comparatively less attention. This systematic review addresses the existing research gap regarding the psychological and social consequences experienced by siblings of individuals with deafblindness. A comprehensive search was conducted across multiple electronic databases, including PsycINFO, PsycARTICLES, Dissertations & Theses (on ProQuest), ERIC (Education Resources Information Center), International Bibliography of the Social Sciences (IBSS), Sociological Abstracts, Google Scholar, PubMed, and Cairn Info. Seven studies were identified as meeting the eligibility criteria for inclusion. The review revealed that siblings of individuals with deafblindness face psychological and social challenges, including emotions such as feelings of neglect, resentment, embarrassment, jealousy, and anxiety. Siblings also grapple with communication difficulties, contributing to feelings of exclusion and insecurity. In addition, these siblings take on significant responsibilities within the family and encounter obstacles in forming relationships outside the family. These findings underscore the need of interventions to improve the well-being of siblings of individuals with deafblindness by addressing their psycho-emotional needs and promoting positive social interactions. These findings align with studies conducted on siblings of children with other disabilities. However, additional research is crucial to investigate overlooked dimensions, particularly positive factors like coping mechanisms and resilience, that may influence the mental health and social experiences of these siblings.

## Introduction

According to Watters et al. (2005), deafblindness is “a condition that combines any degree of hearing loss with any degree of vision loss that interferes with communicating and acquiring information, even though individuals who are deafblind may still have varying levels of useful vision and hearing” (p. 16). Indeed, deafblindness encompasses a spectrum of manifestations (Rodgers, 2021), and most subjects exhibit residual vision and hearing. The first global report of the World Federation of the DeafBlind indicates that approximately 0.2% of the world’s population lives with severe deafblindness, whereas “milder forms” of deafblindness impact around 2% of the global population (International Disability Alliance, 2018). Moreover, individuals with deafblindness frequently exhibit additional physical and cognitive impairments (Heller et al., 1999).

Deafblindness manifests in various forms, including congenital, acquired, and aged-related deafblindness, each with distinct etiological factors and characteristics (Souriau, 2000; Dammeyer, 2010). Congenital deafblindness occurs at birth or shortly thereafter (Dammeyer, 2010) and is caused by genetic factors (e.g.: CHARGE syndrome), prenatal infections (e.g: cytomegalovirus, rubella,), or birth complications (e.g.: as prematurity, low birth weight,). Metabolic disorders, congenital malformations, certain medications, or maternal drug use are other potential causes of congenital deafblindness (Chen, 2004; Holte et al., 2006; National Center on Deaf-Blindness, n.d.).

Acquired deafblindness occurs later in life (following a period of normal sensory functioning) (Dammeyer, 2010) and can be attributed to genetic disorders (e.g.: Usher syndrome), traumatic events (e.g.: severe head injuries), infections (e.g.: meningitis), or specific neuronal conditions (e.g.: multiple sclerosis). Additionally, prolonged use of certain medications or exposure to toxic substances like chemotherapy drugs, antibiotics, or environmental toxins can potentially lead to acquired deafblindness (Chen, 2004; Holte et al., 2006; National Center on Deaf-Blindness, n.d.).

Age-related deafblindness is characterized by a gradual decline of both hearing and vision that arises due to the natural process of aging. This condition predominantly affects older adults, typically occurring after the age of 65. The primary causes of age-related deafblindness include presbycusis (age-related hearing loss), age-related macular degeneration, cataracts, and glaucoma, in conjunction with age-related hearing loss. Furthermore, other age-related health conditions like diabetes, cardiovascular diseases, and neurological disorders can also contribute to hearing loss and vision loss (Simcock, 2017).

Among syndromes associated with deafblindness, CHARGE syndrome is the most frequently encountered. However, in the context of acquired deafblindness, Usher syndrome predominates as the leading cause (Chen, 2004; Holte et al., 2006; National Center on Deaf-Blindness, n.d.). The impact of deafblindness, a distinct condition, is multiplicative (i.e., not simply the sum of vision and hearing impairments) (Fletcher and Guthrie, 2013; Ferrell et al., 2014). For example, individuals with deafblindness cannot compensate for hearing loss through lip reading. Thus, deafblindness is best understood as a precise and individual condition with disabilities distinct from those associated with only vision or hearing impairment (Arcous et al., 2019).

Deafblindness correlates with lower education, increased poverty, and higher unemployment rates (International Disability Alliance, 2018). The age at which the condition starts has a big effect on how difficult it is to cope with, especially when the condition is present from birth, leading to significant limitations (Bruce, 2005; Ronnberg and Borg, 2011). Progressive conditions, like Usher syndrome, bring multifaceted challenges, including access to information, mobility problems, workplace and educational difficulties, social isolation, feelings of insecurity, difficulties in projecting into the future and mental health issues (Arcous et al., 2019). Despite these challenges, appropriate accommodations can mitigate difficulties, which thereby enhances the quality of life for individuals with Usher syndrome (Ellis and Hodges, 2013; Arcous et al., 2019). These accommodations include alternative communication methods, assistive technologies, leisure activities, and social support (given by friends or family) (Arcous et al., 2019).

Indeed, the family plays a fundamental role in the well-being of the person with deafblindness (Spring et al., 2012). The family is the primary source of support (Ellis and Hodges, 2013; Arndt and Parker, 2016). According to Bernard (2013), “The quality of life of deafblind children and adults is greatly influenced by the connection, appropriate support, and interactions with the family” (p. 135). Family members can help with travel, administrative procedures, and daily tasks (Kyle and Barnett, 2012). Family members also play an important role in interpreting and accessing information from the outside world (Kyle and Barnett, 2012; Simcock, 2017). Moreover, the family offers the possibility of social openness (Gullacksen et al., 2011; Arndt and Parker, 2016). Sometimes family members are the only people individuals with deafblindness see during the week. The family can also support self-determination of the deafblind individual (Morgan et al., 2002).

According to Siemon (1984), “Because families are a system, distress in one member affects both the system and each member in it.” (p. 294). It is necessary to acknowledge that the presence of a child with a disability in a family can lead to emotional consequences (felt among all family members), along with additional challenges like increased financial responsibilities and the need for extended caregiving. Families may also experience feelings of grief and loss, which can be amplified based on the severity of the disability (Correa-Torres, 2008). Family members may experience both social and psychological consequences due to the presence of a child with a disability.

Social consequences pertain to the effects and repercussions on individuals’ social interactions, relationships, and integration within society, stemming from particular circumstances, events, or conditions. These consequences encompass strained social dynamics, social exclusion or isolation, challenges in forming and maintaining relationships, stigma or discrimination, and difficulties in engaging in social activities or fulfilling social roles (Brooks, 1980; Mormiche and Boissonnat, 2003). Psychological consequences encompass the effects and influences on individuals’ emotional, cognitive, and behavioral well-being from experienced circumstances or events. These consequences encompass emotional responses such as stress, anxiety, depression, anger, sadness and cognitive changes (Jover, 2014; Ha Namkung and Carr, 2020).

Regarding the social consequences, the presence of an individual living with deafblindness poses communication challenges within the family (Souriau, 2000; Rodgers, 2021). Indeed, a study conducted by Kyle and Barnett (2012), among 39 individuals with acquired deafblindness, demonstrated that family members may encounter difficulties acquiring tactile sign language or other specific communication skills tailored to the disability. It is thus evident that effective communication within the family plays a pivotal role in managing the disability, fostering the self-esteem of the individual with a disability, and promoting family cohesion (Miner, 1995; Wahlqvist et al., 2020).

When deafblindness is acquired progressively, this process can provoke significant familial disruptions, which in many cases leads to entire role realignments assumed by family members (Gullacksen et al., 2011)— a process that is neither straightforward nor undemanding. For instance, according to Watters-Miles (2014), who conducted a study among six individuals with Usher syndrome, parents may become overprotective of their children. Hersh (2013) study on individuals with deafblindness (unspecified type) similarly observes protective behaviors demonstrated by parents. Miner (1995) study, focusing on individuals with Usher syndrome type I, emphasizes the need for adjustments in familial relationships as the syndrome progresses. Similarly, Figueiredo et al. (2013) study, involving eleven individuals with Usher syndrome (type I or II), supports Miner’s study results.

The presence of a child with deafblindness can moreover present challenges in socialization beyond the immediate household for family members. A study conducted by Hersh (2013), which examined twenty-seven individuals with deafblindness (without specification of the type), revealed that families with children with deafblindness might experience feelings of stigmatization and embarrassment, ultimately leading to adverse consequences for their social participation.

Regarding the psychological consequences, Watters-Miles (2014) emphasizes that the onset of a disability can be traumatic for the individual and the entire family. Miner (1995), focusing on individuals with Usher syndrome type I, highlights that communication difficulties, hindered by the nature of the disability, can result in additional stress and can increased risk of depression among family members. Indeed, according to Correa-Torres (2008), sighted-hearing parents of children with deafblindness are at a higher risk of developing depression. Hartshorne and Schmittel (2016) who conducted a study on the social–emotional development of children with deafblindness (all types) also found that the risk of sighted-hearing parents developing depression is greater. Emotional problems were also reported by partners of individuals with Usher type 1. These partners appear to have more daily responsibilities and feel tired, depressed and resentful (Miner, 1995).

In summary, the familial implications of deafblindness are both pervasive and multidimensional, extending to siblings who have been relatively overlooked in current literature. Addressing these gaps is vital for the inclusive support and well-being of all family members. Indeed, the siblings of children with deafblindness confront unique challenges due to the very nature of their brother or sister’s disability. Because of visual and auditory impairment, siblings are greatly hindered in their ability to communicate and play with their disabled sibling.

Siblings also assume increasingly vital roles within the family, often enduring lifelong relationships with their disabled siblings (Cicirelli, 1995). Indeed, siblings share a deep and enduring history together. As parents age, siblings may take on greater responsibility and play a more meaningful role in their relationship with a sibling with a disability (Vert et al., 2016). Also, siblings serve as primary witnesses to the challenges faced by the disabled child and their parents (Dayan and Scelles, 2017).

Despite this, research has primarily concentrated on parents and spouses, leaving a gap in understanding the psychological and social experiences of siblings of children with deafblindness.^1^ To bridge this gap in knowledge, this systematic review aims to explore the existing literature on the psychological and social consequences of deafblindness on siblings. Gaining a comprehensive understanding of their experiences is crucial for providing inclusive support and enhancing the overall well-being of the entire family.

## Methodology

This systematic literature review was carried out to explore the existing literature on the psychological and social consequences of deafblindness on siblings.

### Search strategy

We conducted a comprehensive literature search using various electronic databases, including PsycINFO, PsycARTICLES, Dissertations & Theses (on ProQuest), ERIC (Education Resources Information Center), International Bibliography of the Social Sciences (IBSS), Sociological Abstracts, Google Scholar, PubMed, and Cairn Info. The following combination of words was used to conduct the systematic review search: “Siblings” OR “Brother,” OR “Brothers” OR “Sister” OR “Sisters” OR “Fratrie” OR “Frère” OR “frères” OR “sœurs” OR “Soeur” AND (“Sourdaveugle” OR “Sourd-aveugle” OR “Surdicécité” OR “Deaf-blind” OR “Deafblind” OR “Deafblindness” OR “Dual Sensory loss” OR “dual sensory impair” OR “Dual sensory impairment” or “Dual sensory disability” OR “Usher Syndrome” OR “Wolfram Syndrome” OR “Stickler Syndrome” OR “Charge Syndrome” OR “Alport Syndrome” OR “Bardet-Biedl Syndrome” OR “Rubella” OR “Cockayne Syndrome” OR “Cornelia de Lange Syndrome” OR “Flynn-Aird Syndrome” OR “Goldenhar Syndrome” OR “Deaf-blind Hypopigmentation Syndrome”).

### Inclusion and exclusion criteria

Studies were included in the review if they were (1) written in English or in French languages (two languages spoken by the authors), (2) published in a peer-reviewed journal/ published dissertation/thesis studies or published reports with no date limits for the publication year (3) explored life experience and/or psychological health and/or social experience of siblings of individuals with deafblindness, and (4) articles mentioning the terms “fratrie” (siblings), “siblings,” “frères” (brothers), “frère” (brother), “sœurs” (sisters), “sœur” (sister).

Studies were excluded from the review if they primarily focused on age-related deafblindness. This exclusion criterion was implemented to ensure that the included studies specifically addressed the experiences of siblings who grew up in the same household as individuals with deafblindness during childhood or adolescence.

### Date selection and collection process

The articles selection process was conducted by two authors (first and second author). Here are the 6 steps that we followed for the article selection: (1) Database Retrieval; (2) Duplicate Removal; (3) Title and Abstract Screening; (4) Accessibility Check; (5) Criteria-Based Selection; and (6) Final Selection.

1. Number of articles obtained in each database: the number of articles collected from each database was recorded to determine the initial pool of resources available for analysis. One thousand six hundred fourteen (1614) articles were found in total in the databases.
2. Number of articles set discarded as duplicates: to ensure data integrity and avoid redundancy, duplicate articles across databases were identified and removed from the pool. This step helped streamline the analysis process and eliminate any repetition. Two hundred and seventeen duplicates were identified, and 1,397 articles remained after removing these duplicates.
3. Number of articles set aside based on titles and abstract: after obtaining the articles (1,397 after removing the duplicates), a screening process was conducted based on their titles and abstracts. Articles that did not appear relevant to the research topic or did not meet the inclusion criteria were discarded. Many articles discussed the chosen syndromes or the chosen syndromes in relation to siblings. However, most of these studies had a medical perspective, aiming to uncover the genetic factors behind disease transmission rather than exploring the personal and social experiences of the siblings. One thousand three hundred fifty-one (1351) were thus rejected at this step in our methodology. Eighteen articles remained.
4. Among the eighteen remaining articles, we excluded some due to accessibility issues. In cases where we could only access the title or the abstract without the full text, we left these articles out of our analysis. This was done to ensure a thorough analysis, focusing on complete articles rather than just titles or abstracts. Specifically, we excluded two articles because we could only retrieve their titles, and four more because we had access only to their abstracts.
5. Number of articles selected for analysis: from the remaining articles, a selection was made based on the predetermined criteria for inclusion. These criteria could include relevance to the research question or methodology problems. Twelve articles were screened for eligibility using the inclusion and exclusion criteria. Five articles were excluded.
6. Final number of articles selected: after screening and applying the inclusion criteria, the remaining articles constituted the final set of resources selected for analysis. The number of articles (7) at this stage represents the data used in the research study.

### Data extraction

**Figure 1:**
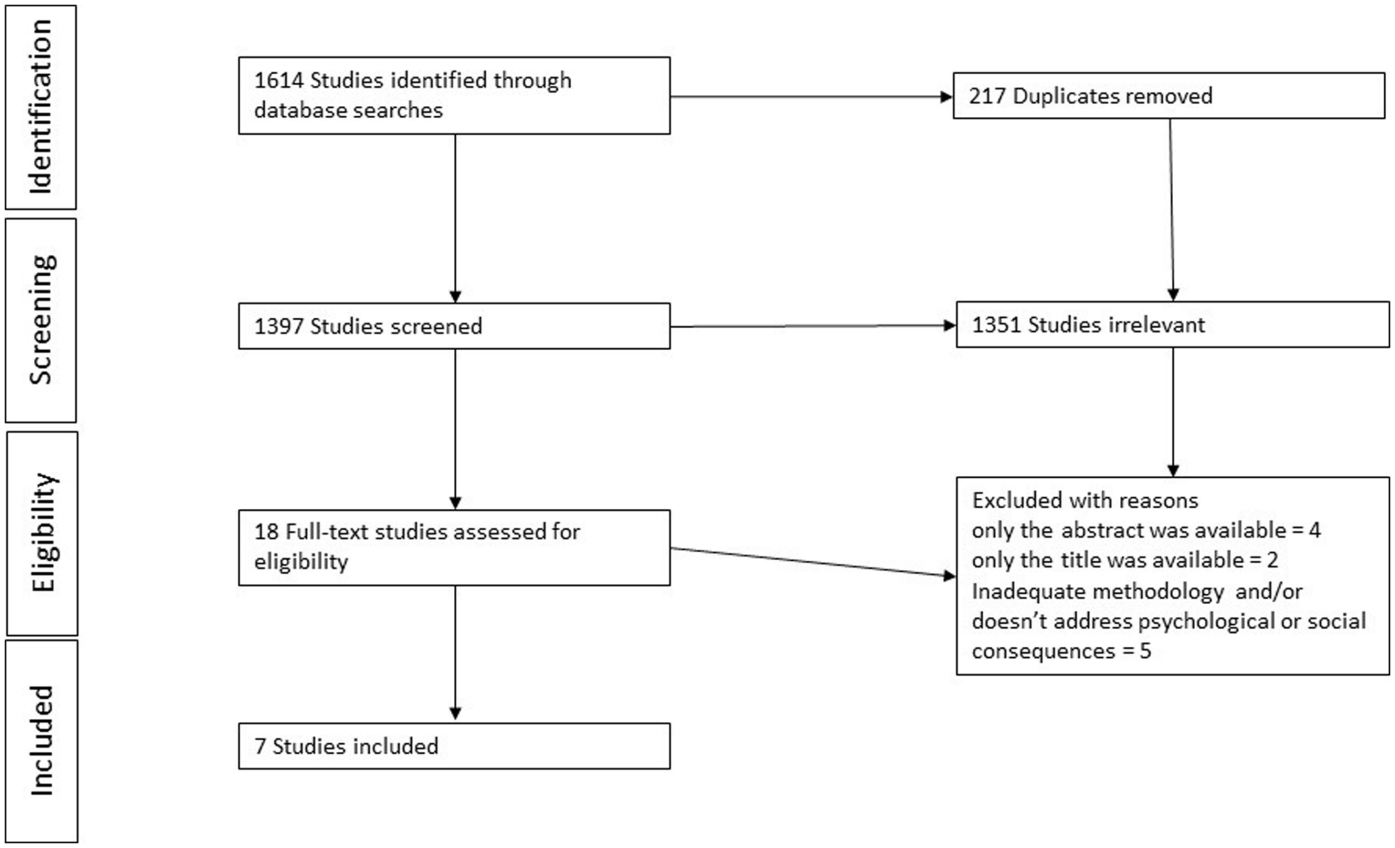
Data selection process.

The two authors extracted the selected articles based on their relevance to the inclusion criteria. One author was responsible for categorizing the results into psychological and social consequences, utilizing the definitions provided in the introduction. The second author validated this categorization. Information about the country the research was carried out, the age of the siblings, the type of deafblindness of the siblings, the methodology used in the articles and the bias and limitations presented by the authors of each article were also gathered (Figure 1).

## Results

### General characteristics of the studies

In total, there were five studies conducted in the United States, 1 study in Australia, 1 study in Canada, and 1 study in the United Kingdom. Only four studies directly assessed siblings’ experiences with their siblings with deafblindness. The other three studies examined siblings’ experiences through the perspectives of parents or individuals living with deafblindness.

Rowan (1990) conducted a study in the United States involving 12 siblings of deafblind children from five families. The age range of the siblings in this study was between 7 and 16 years old. Another study in the United States by Laman (2006) focused on four siblings ranging in age from 19 to 22 years old, with a sibling with congenital deafblindness. Harland and Cuskelly (2000) conducted a study in Australia with four siblings from different families. The age range of the siblings in this study was between 21 and 30 years old.

The causes of deafblindness in siblings with this disability were different. We believe it is necessary to clarify this because the experiences of non-disabled siblings may vary depending on the severity of the visual and hearing impairment. Rowan (1990) discussed different conditions, such as cortical visual impairment, profound hearing loss, and associated complications. Laman (2006) focused on congenital deafblindness without specifying the cause. Harland and Cuskelly (2000) explored cases of moderate to profound vision and hearing impairments, often accompanied by intellectual and physical disabilities. Vert et al. (2016) specifically studied individuals with CHARGE syndrome. Watters et al. (2005) investigated both acquired and congenital forms of deafblindness. Ellis and Hodges (2013) examined Usher syndrome, distinguishing between type I, type II, and type III. Finally, Heller et al. (1999) study focused on children with deafblindness without specifiying the various types.

Several studies have adopted qualitative methods to explore siblings’ experiences in the context of deafblindness. Rowan (1990), a graduate student in education, conducted one-on-one interviews with siblings to gain insights into their perspectives. Laman (2006), a doctor in special education, employed semi-structured interviews to examine adult siblings’ perceptions of their involvement in the Individualized Transition Plan (ITP) of siblings with congenital deafblindness. Harland and Cuskelly (2000), two researchers in special education, conducted semi-structured interviews on sibling responsibilities, support, personal development, advocacy, and more topics. Watters et al. (2005), researchers in disability studies, conducted focus groups with individuals with deafblindness, parents/advocates, and interviews with service providers. Ellis and Hodges (2013), researchers in education, employed semi-structured and extensive interviews.

In addition to qualitative approaches, quantitative studies have examined siblings’ experiences with deafblindness. Vert et al. (2016) utilized the Sibling Evaluation Questionnaire, UCLA Loneliness Scale, Network Orientation Scale, Family Hardiness Index, and Family Member Well-Being Index to assess various sibling experiences and well-being aspects. Furthermore, Heller et al. (1999) used a questionnaire to evaluate parents’ perceptions of siblings’ interactions with their brothers and sisters with deafblindness (Table 1).

**Table 1:** Characteristics of the studies.

Authors/date	Country	Type of publication	Type of study
Rowan (1990)	USA	Master dissertation in Education	Qualitative And Quantitative
Heller et al. (1999)	USA	Peer-reviewed article	Quantitative
Watters et al. (2005)	Canada	Report from the Canadian Society of deaf-Blind	Qualitative and Quantitative
Laman (2006)	USA	Dissertation in Special Education for the degree of Doctor of Education – Faculty of Texas Tech University	Qualitative
Harland and Cuskelly (2000)	Australia	Peer-reviewed article	Qualitative
Ellis and Hodges (2013)	U.K	Research report University of Birmingham	Qualitative and Quantitative
Vert et al. (2016)	USA	Peer-reviewed article	Quantitative

### Quality of the study

Several limitations and biases have been identified in the examined studies by the authors of each study themselves, highlighting the importance of considering these factors when interpreting the findings. As an example, an interview may lead to the unintended procurement of socially desirable responses, especially when it is based on voluntary participation (Rowan, 1990; Harland and Cuskelly, 2000; Laman, 2006). Also, as Rowan (1990) mentioned, the understanding of questions by siblings and the author’s interpretation could influence results.

Small sample sizes from specific regions limit the generalizability of the findings (Rowan, 1990; Heller et al., 1999; Harland and Cuskelly, 2000; Laman, 2006; Vert et al., 2016). Additionally, three studies did not directly assess the siblings experience but investigated people with deafblindness’ perspective of their siblings’ experience (Watters et al., 2005; Ellis and Hodges, 2013) or parents’ perspectives (Heller et al., 1999) (Table 2).

**Table 2:** Characteristics of the studies.

Authors/date	Participants	Methodology	Limitations/bias
Rowan (1990)	12 siblings of deafblind children from five families in the Utah Intervener Services Program. 8 males and 4 females, ages ranging from 7 to 16 years old. Deafblind children: 2 males and 3 females, ages ranging from 1 year 10 months to 3 years 5 months. Deafblind children’s conditions: failure to thrive, cortical visual impairment, profound hearing loss; cortical visual impairment, mild hearing loss; cortical visual impairment, cortical hearing impairment; severe handicaps, cortical visual impairment, cortical hearing impairment; profound hearing loss, retinopathy of prematurity, exotropia of the right eye. Additional complications: Many of the deaf-blind children had complications such as requiring oxygen and tube feeding, and some had seizures.	Qualitative: one-on-one interviews with the siblings. Quantitative: The Siblings’ Perceptions of the Intervener Interview (SPII) and Taylor’s Siblings’ problems Questionnaire were administered.	Voluntary participation, potentially biased towards individuals with positive attitudes. Small sample size from only one state, limiting generalizability to other siblings of deaf-blind children. The assessment tools used lacked reliability and validity information. Only one interview may have resulted in socially desirable responses. Siblings’ understanding of questions and the author’s interpretation could influence results.
Heller et al. (1999)	36 parents of children with deafblindness. Ranging age of the children 1 to 22 years old. A sibling is living at least at home. Ranging age of the siblings (1 to 22 years old). Type of disability: Congenital deafblindess. Most participants’ children have additional physical complications, such as orthopedic impairments, health impairments, and mental retardation. Most siblings were older than the child with deafblindness (61.1%). Siblings gender distribution: Almost an even distribution by gender for both the deaf-blind children and their siblings. Deaf-blindness causes: Majority of children had deaf-blindness due to multiple congenital anomalies (Charge syndrome or Rubella was the most present). Additional disabilities: 92% of children with deafblindness had additional disabilities, including orthopedic impairments, health impairments, and mental retardation.	Questionnaire to evaluate parents’perceptions of siblings’interactions with their brothers and sisters who are deafblind.	Smal sampel size. Only parents perception and not siblings’s one.
Watters et al. (2005)	44 participants with deafblindness (29 females and 15 males). Age ranging from 20 to 75 years old. 42 respondents with acquired deafblindness and 2 respondents with congenital deafblindness. Focus group with parents/advocates: Date of the focus group is not available. Interviews with service providers: Data from the interviews are not available.	Quantitative: Questionnaire to gather demographics data (through telephone/emails) Qualitative: Focus groups with deafblind people or parents/advocates Service provider interviews	The reported experiences are not directly from siblings but from individuals living with deafblindness. Number of parents/advocates who participated to the focus group is unspecified. Number of service providers who participated to the interviews is unspecified Limited to Canada.
Laman (2006)	4 siblings, age ranging from 19 to 22 years old. Who have a sibling with congenital deafblindness (cause undefined)	Semi-structured interviews	Voluntary participation, potentially biased towards individuals with positive attitudes. Small sample size from only one state, limiting generalizability to other siblings of deaf-blind children. Only one interview may have resulted in socially desirable responses. Siblings’ understanding of questions and author’s interpretation could influence results.
Harland and Cuskelly (2000)	4 siblings from different South East Queensland, Australia families participated in the study. Age ranging from 21 to 30 years old. Type of disability: moderate to profound vision and hearing impairments, and in some cases, additional intellectual and physical disabilities.	Semi-structured interviews covering various topics, including responsibilities, support, development, and suggestions for improving quality of life. Grounded theory was used as the research approach.	Only one sibling per family was interviewed. Voluntary participation of siblings may introduce bias. The most supportive sibling may have been selected by the parent in families Siblings’ biases towards the researcher and interpreter may affect validity. Small sample size. Cultural differences may impact the transferability of disability impact studies.
Vert et al. (2016)	29 siblings of children with charge syndrome. Age ranging from 13 to 42 years old. 14 males and 15 females. Severity of CHARGE syndrome: Majority (69%) of individuals with CHARGE were rated as moderately affected, with a minority being mildly or severely affected.	Sibling Evaluation Questionnaire (SEQ) UCLA Loneliness Scale (LS) Network Orientation Scale (NOS) Family Hardiness Index Family Member Well-Being Index	CHARGE research may not apply to other syndromes. Severity of disability affects sibling adjustment. Small sample size Lack of sibling relationship measures, SEQ validation needed. Siblings may show positive bias, influencing results.

### Psychological consequences

Siblings may experience certain negative emotions such as resentment towards their parents, primarily due to the parents’ reduced physical and emotional availability (Watters et al., 2005). Some siblings experienced feelings of exclusion and jealousy due to the additional parental attention given to the child with deafblindness (Watters et al., 2005). For example, in the study conducted by Ellis and Hodges (2013), Bethany (17, type 2) shared her experience of being perceived as the “favorite” among her siblings because she received more attention, which created tension with her sibling. In contrast, another study by Rowan (1990) revealed that nine out of twelve siblings did not perceive their siblings with deafblindness as more fortunate due to the attention or special treatment’ received by the child with deafblindness.

Moreover, in Harland and Cuskelly (2000) study, some siblings expressed guilt over their inability to take a more active role in caring for their disabled sibling. They recognized the burden of care placed on their parents but felt helpless in changing the situation. In this study, one sibling expressed his guilt by saying, “I wish I could do more for Mum because she does do an awful lot.” (p. 300). The same authors noticed that some siblings encounter feelings of anxiety as they grapple with understanding and managing their disabled sibling’s behavior. Interacting with a sibling who has disabilities can indeed present challenges, potentially leading to anxiety and frustration for siblings (Harland and Cuskelly, 2000). Communication barriers, personal insecurities, and the uncertainty surrounding their sibling’s receptiveness to communication may contribute to feelings of exclusion and insecurity (Harland and Cuskelly, 2000).

Also, siblings may experience a sense of inequity and frustration because of the family’s financial problems that are caused by having a child with deafblindness (Watters et al., 2005). Additionally, in Rowan study’s (1990), some siblings mentioned that they felt they bore additional responsibilities which caused frustration and a sense of unfairness. In Rowan study’s, some siblings (3 out of 12) stated they put pressure on themselves to succeed academically or in other areas to compensate for the limitations of their sibling with deafblindness. This self-imposed pressure could contribute to their anxiety. Social embarrassment due to friends’ reactions and questions further exacerbated the emotional toll (Rowan, 1990).

Anxiety may also arise from difficulty envisioning the future. Indeed, sibling disability can significantly impact an individual’s perception of their future, as they envision a lifelong role in the life of the child with deafblindness (Harland and Cuskelly, 2000; Laman, 2006). In Laman (2006) study, all the siblings expressed concerns and anxieties regarding their future roles once their parents could no longer support the person with disabilities. Those results are supported by Harland and Cuskelly (2000) results. In Harland and Cuskelly’s study, some siblings also expressed anxiety and concerns for the future. The siblings recognized the importance of future financial support. They acknowledged that their roles would need to evolve as their parents could not provide primary home-based care for their siblings. Furthermore, many siblings expressed inadequacy in not being able to assume greater responsibility for supporting their siblings. This sense of inadequacy was influenced by various factors, resulting in stress and conflict among most siblings (Harland and Cuskelly, 2000). Time constraints emerged as a concern for five out of twelve of the siblings in Rowan (1990). They expressed worries about having limited time to support their siblings with disabilities due to their numerous other commitments.

Furthermore, some siblings experienced deep distress regarding their lack of preparedness for their future responsibilities towards their sibling with deafblindness. Despite the anxiety stemming from their parents’ mortality, all the siblings were willing to assume increased responsibility if necessary to support their siblings with disabilities (Harland and Cuskelly, 2000). In Harland and Cuskelly (2000) study, most siblings were expected to continue their responsibility for supporting their sibling’s personal development. However, some siblings, like Phillip (a sibling interviewed by Harland and Cuskelly, 2000), doubted their ability to take on teaching responsibilities themselves.

The search for suitable accommodation for their sibling with deafblindness was identified as a significant concern for some siblings. In Harland and Cuskelly (2000) study, one sibling stated, “There is nothing for the deafblind in the area of job preparation and placement” (p. 302). Another sibling was concerned about her ability to find appropriate accommodation for her sister in the future. Some siblings also expressed anxiety about the level of care their brother or sister would receive in non-family-based supported accommodation settings (Harland and Cuskelly, 2000). Assisting with or managing their sibling’s financial affairs was another responsibility that most siblings anticipated undertaking in the future (Harland and Cuskelly, 2000). Rowan (1990) stated that the worries and concerns expressed by some siblings of children with deafblindness encompassed fears, concerns, worries and anxieties about their sibling’s mobility, mortality, future employment prospects, and access to education (Rowan, 1990).

Harland and Cuskelly (2000) noticed that some siblings expressed previous apprehensions about the potential of having children with disabilities themselves. One participant in their study sought genetic counseling to address this concern, and another one worried that prospective partners might mistakenly assume genetic complications associated with their relationship.

The experience of having a sibling with deafblindness can give rise to a strong need for information among siblings, leading to confusion, incomprehension, and anxiety. Understanding the specific problems faced by the child with deafblindness is another significant concern for siblings. Some siblings expressed desire to gain deeper insights into terminology, causes of the handicap, functioning abilities, caregiving techniques, preferences, and future prospects for their sibling (Rowan, 1990). They may desire to understand the nature and extent of their sibling’s disability in hopes of finding a sense of clarity about what it means to be deafblind (Rowan, 1990). They questioned whether their sibling is truly deaf, blind, or both, highlighting the need for accurate information to dispel misconceptions (Rowan, 1990). In addition to seeking information, siblings recognized their responsibility as advocates for their siblings with deafblindness. They strongly desired to learn more about available services and opportunities, understanding that this knowledge is crucial for their future role. However, the lack of information leaves them uncertain about their sibling’s future plans and legal matters, causing anxiety and a need for clarity (Harland and Cuskelly, 2000). Concerns about future living arrangements and the potential changes in their sibling’s condition further contribute to siblings’ anxiety. Some siblings believed that their sibling with deafblindness may not always live at home, while others believed their sibling’s deafblindness may change over time (Harland and Cuskelly, 2000). In Heller et al. (1999) study, 67% of the parents recognized the siblings’ desire to learn more about interacting and communicating with their sibling with deafblindness, emphasizing the importance of knowledge and understanding in facilitating meaningful connections.

Banta (1979) highlighted the siblings’ greater distress compared to their parents as a result of ineffective coping mechanisms. As Harland and Cuskelly (2000) noticed, siblings relied heavily on their parents for help and guidance in caring for their sibling with deafblindness. They sought assistance in understanding the special attention and care required by a child with deafblindness, learning how to use specialized equipment, effectively communicate, handle emotional situations, and provide proper nourishment (Harland and Cuskelly, 2000). To cope with their worries and problems, siblings actively sought emotional support from various sources. Mothers are often their primary confidants, followed by fathers and friends (Rowan, 1990) (Table 3).

**Table 3:** Results.

Authors/date	Psychological consequences	Social consequences
Rowan (1990)	Seek information on deafblindness. Feel embarrassed and perceive adverse reactions from friends. Experience frustration, anxiety, and early responsibilities with pressure to succeed. Bear additional household duties, leading to frustration and unfairness. Express guilt over limited involvement in caring for their disabled sibling. Have concerns about future roles without parental support. Recognize the importance of emotional support from parents and friends Do not perceive their sibling as fortunate or receiving special treatment.	Take on various responsibilities within the family dynamics to care for a deaf-blind individual. Caregiver and protectors. Possess knowledge and skills to assist the deaf-blind individual. Seek information on services, living arrangements, and the condition of the deafblind individual.
Heller et al. (1999)	Feel anxious about the future.	Adopt an helping role. Seek support for communication with their deafblind sibling. Limited interaction or avoidance may occur. Adjustments for participation may be lacking. Communication challenges hinder interaction. Nonsymbolic communication is common. Communication adjustments are necessary.
Watters et al. (2005)	Resentment towards parents and the deafblind child Experience negative emotions like exclusion, jealousy, and resentment. Bearing financial responsibility for their activities may cause a sense of inequity and frustration. Perceive it as unfair and straining the family’s limited resources.	Face difficulties in interacting with their deafblind sibling due to communication challenges. Communication adjustments and adaptations, including augmentative and alternative communication methods.
Laman (2006)	Anxiety and uncertainty about what is expected from them in the future.	Want to be involved in their sibling’s future.
Harland and Cuskelly (2000)	Interacting with a sibling with disabilities presents challenges and causes anxiety and frustration. Siblings express a need for information and understanding to support their siblings. Express guilt over their inability to take a more active role in caring Experience anxiety in managing their sibling’s challenging behavior. Concerns arise about their sibling’s future plans, legal matters, living arrangements, changes in condition, and employment prospects. Stress and conflict arise due to feelings of inadequacy and time constraints. Siblings feel unprepared for future responsibilities and doubt their ability to assume teaching roles. Have apprehensions about having children with disabilities themselves. Emotional support from parents and friends is recognized as essential and sought after.	Face challenges and disadvantages, Encounter attention and stares from others, limited space, and disrupted routines. Expressing dissatisfaction, they mention the interference of their deaf-blind sibling in their lives and plans. Embrace a helping role.
Ellis and Hodges (2013)	Jealousy felt by siblings towards the deafblind child. Perceive favoritism among their siblings, as they receive more attention from adults.	In need of support and advice from specialist services and networks who understood Usher syndrome better.
Vert et al. (2016)	Rate their personal well-being positively. Siblings’ relationship are associated with hardiness, loneliness, and Charge knowledge.	Accept their sibling with Charge syndrome neutrally. Report less loneliness, and similar social support access. Benefit from interacting with other children with disabled siblings.

### Social consequences

Siblings may experience difficulties in interaction with their deafblind brother or sister. Indeed, the presence of communication difficulties related to the nature of the disability, which affects language, makes it difficult for siblings to engage in activities with their sibling with deafblindness (Heller et al., 1999; Watters et al., 2005). Communication is an important aspect of the sibling relationship that often needs adjustments and adaptations, commonly achieved through augmentative and alternative methods of communication. In the case of siblings with a brother or sister with deafblindness, it has been observed that they mostly rely on nonsymbolic forms of communication to interact. However, there is a noticeable absence of alternative communication methods, such as tactile or visual communication boards/systems, and limited involvement from professionals in teaching siblings how to communicate with their sibling with deafblindness. Interestingly, most siblings acquired nonsymbolic communication skills independently, as reported by 62.5% of parents and 56.3% of parents of elementary school children (Heller et al., 1999). On the other hand, sign language is predominantly taught by a parent/relative or, in some instances, by a speech therapist, as seen with two older children. Among parents who reported that siblings face communication challenges consistently, those who rely on nonsymbolic forms of communication experienced the greatest difficulty. Additionally, it seems that the nature of these difficulties is influenced by the severity of the impairment and the availability of alternative communication methods (Heller et al., 1999).

In Heller et al. (1999) study, most parents (over 25%) reported that siblings have minimal interaction or deliberately try to avoid their brother or sister with deafblindness. Many parents (22.2%) and parents of elementary school children (16.7%) indicated that siblings never try to engage their brother or sister in any activity. Regarding the duration of sibling engagement, approximately 23.5% of parents and 21.7% of parents of elementary school children reported a daily involvement of 5–15 min, while 20.6% of parents and 26.1% of parents of elementary school children reported spending 30 min to an hour together. However, a notable percentage of parents (14.7%) and parents of elementary school children (21.7%) mentioned that siblings spend less than 5 min with their sibling with deeafblindness, and 17.6 and 8.7% of parents and parents of elementary school children, respectively, stated that no dedicated time is spent between siblings. The frequency of difficulty encountered in playing or engaging in activities together varied among respondents. The majority of parents (44.4%) and parents of elementary school children (41.7%) reported that such difficulties occurred some of the time. When questioned about the siblings’ willingness to make necessary adjustments for their brother or sister with deafblindness to participate in games or activities that require visual perception, a significant percentage of parents (68.6%) and of parents of elementary school children (70.8%) indicated that siblings never made adaptations or only did so on certain occasions. Similarly, for activities requiring hearing, most parents (70.6%) and parents of elementary school children (73.9%) reported that siblings never made accommodations or only did so sometimes.

According to Rowan (1990), siblings of individuals with deafblindness play pivotal roles in family dynamics, specifically in caring for and supporting their sibling with deafblindness. In Rowan (1990), a majority (eight out of twelve) indicated that their parents expected them to take on additional responsibilities due to the presence of a sibling with deafblindness. These responsibilities span from babysitting their deafblind sibling and other siblings, assisting younger siblings with chores, bathing, feeding, changing diapers, dressing, providing tracheostomy care, engaging in playtime, attending to the child’s needs during the night, cooking, and undertaking household to outdoor tasks. This underscores the heightened responsibility these siblings’ experience, with two of the twelve respondents citing exclusive household roles arising from their deafblind sibling’s needs (Rowan, 1990). Additionally, six of eleven siblings noted their caregiving role was more extensive than their peers’ involvement with their own siblings. However, ten out of twelve siblings perceive their parents’ rules as fair, indicating a balanced approach to managing family dynamics. Concerning knowledge and skills, six out of twelve siblings reported being adequately equipped to assist their sibling with deafblindness (Rowan, 1990).

In Heller et al. (1999) study, parents characterized the relationship between siblings and the child with deafblindness as “helping.” The study revealed that while mothers were the primary caregivers, siblings played a significant albeit secondary role in supporting their brother or sister with visual and hearing impairments. Harland and Cuskelly (2000) found in their study that most siblings provided practical assistance to their brother or sister with deafblindness (e.g., in mobility, recreational activities, and respite care). In some cases, they even took on the role of being a parent for two weeks to give their parents a break (Harland and Cuskelly, 2000). Most siblings offered practical assistance by reinforcing or teaching their brother or sister with deafblindness new skills. One common responsibility shouldered by adult siblings was maintaining regular contact with their siblings with disabilities. In fact, adult siblings maintained regular contact and emotional support, reinforcing or teaching new skills *regardless* of geographic proximity (Harland and Cuskelly, 2000).

Siblings of children with deafblindness encounter various challenges and disadvantages in their everyday lives. Public attention when accompanying their sibling with deafblindness and crowded living conditions due to special equipment are among the drawbacks (Rowan, 1990). Frequent hospital visits further add to the siblings’ inconvenience and disruption of their personal routine. Another disadvantage is the impact on their participation in family activities, as their sibling’s condition may require adjustments or prevent them from fully engaging in shared experiences (Rowan, 1990). In Rowan (1990), nine out of twelve siblings expressed dissatisfaction with how their brother or sister with deafblindness interfered in their lives. This dissatisfaction was often associated with the inability to implement family plans and activities due to their sibling’s needs (i.e., a parent staying home with the child with deafblindness rather than joining the rest of a family on an outing). Furthermore, four out of ten siblings shared their discontent with their sibling’s impact on their personal plans and activities. These concerns range from being unable to visit friends’ houses or host them at their homes, feeling obligated to be involved with the care of their sibling with deafblindness, and having their activities disrupted due to the responsibility of looking after their other siblings. To address the limited participation in activities, it is recommended, within the literature, to modify the activities to accommodate the vision and hearing loss of the child with deafblindness. This modification can enhance siblings’ ability to participate in and foster meaningful interaction. However, it is worth noting that siblings may lack information on how to effectively modify or identify suitable activities for their siblings with deafblindness (Rowan, 1990).

Rowan (1990) also notes the generally positive and protective attitudes of siblings towards their sibling with deafblindness. Notably, in Rowan (1990) all siblings demonstrated a favorable attitude and actively discouraged any teasing directed towards their sibling with deafblindness. Despite the challenges they may encounter, siblings cited genuine enjoyment/happiness stemming from their relationship with their brother/sister with deafblindness and are committed to safeguarding their well-being when necessary. Among the surveyed, six out of the twelve siblings mentioned that their friends visit their home and engage in various activities, such as playing pat-a-cake, throwing a ball, holding hands, and communicating with the child with deafblindness. On the contrary, six siblings reported that their friends do not come to their homes to interact with their siblings with deafblindness. When explaining their sibling’s condition to friends, five out of the twelve siblings expressed no difficulty conveying the necessary information. Feelings of rejection were minimal (eight out of twelve), with most embracing their sibling’s condition openly, showing unconditional love and acceptance. Several stated they did not wish for their deafblind sibling to be absent, highlighting the depth of their emotional bond (Rowan, 1990).

However, Vert et al. (2016) study found a correlation between siblings’ level of difficulty in engaging with their sibling with deafblindness and their willingness to include them in activities with friends or attend support groups. This suggests the potential benefit of interaction with peers who also have siblings with disabilities (Table 3).

## Discussion

While studies have explored the experiences of siblings of individuals with disabilities, the literature on siblings of those with deafblindness remains notably sparse. The existing research does, however, illuminate the unique psychological and social challenges these siblings face.

To summarize, the psychological consequences experienced by siblings of deafblind individuals can include a range of negative emotions such as resentment, jealousy, frustration, guilt, anxiety, and feelings of inadequacy. Siblings may feel resentment towards their parents due to reduced attention and availability, as well as jealousy towards the additional parental attention given to the deafblind child. They may also feel excluded and experience a sense of inequity and frustration. Siblings often express guilt over their inability to take a more active role in caring for their disabled sibling and may experience anxiety when managing their sibling’s challenging behavior. Communication barriers, personal insecurities, and uncertainty about their sibling’s receptiveness to communication can contribute to feelings of exclusion and insecurity. Siblings may also experience anxiety when envisioning the future, particularly regarding their own future roles, once their parents can no longer provide support for the person with deafblindness.

Social consequences for siblings of individuals with deafblindness can include difficulties in interacting with their siblings due to communication challenges related to the disability. Siblings often rely on nonsymbolic forms of communication to interact. Still, there may be a lack of alternative communication methods and limited involvement from professionals in teaching siblings how to communicate with their siblings with deafblindness. Siblings also take on significant responsibilities within the family dynamics, such as caregiving tasks and support for their sibling with deafblindness which impact the time they have for their social activities. In the context of family activities/outings, siblings may experience disadvantages, such as attention and stares from others, routines disruptions, and limited household space. Siblings may also encounter difficulties forming and maintaining relationships outside the family due to their sibling’s condition. Some siblings may actively avoid or have minimal interaction with their deafblind sibling.

Similar experiences have been observed in siblings of children with other types of disabilities. For example, some studies on individuals with undefined physical or chronic disabilities demonstrated that siblings of children with disabilities can experience distress, unhappiness and resentment (Lamarche, 1985; Fisman et al., 2000; Scelles, 2011; Giallo et al., 2012; Hallberg, 2013).

Also, siblings of children with other disabilities (undefined physical or intellectual disabilities) can feel ashamed of how others perceive them and struggle with social inclusion (Lamarche, 1985; Dayan and Scelles, 2017).

Siblings of children with other disabilities (hemiparesis and undefined physical or intellectual disabilities) may exhibit premature and hyper-protective behaviors, feel responsible for the family’s well-being, and seek partners who understand their role (Seligman, 1983; Hannah and Midlarsky, 1985; Scelles, 2004; Gardou, 2012; Dufreche Rastello, 2020).

In addition, siblings of children with other disabilities (undefined chronic illness) tend to have higher anxiety levels stemming from uncertainty in interacting with the disabled sibling (Heiney et al., 1990; Martinez et al., 2022).

Finally, siblings of children with hemiparesis express the same lack of information about the disability (Dufreche Rastello, 2020), as for siblings of individuals with deafblindness. Research indicates that siblings of children with hemiparesis desire more information but fear the implications it may have on themselves (Dufreche Rastello, 2020). Limited contact with professionals and guilt often prevents these siblings from asking questions that might equip them with the information they desire, such as questions that address the nature of their siblings disability (Smith and Perry, 2005).

In contrast, some aspects present in the siblings of children with disabilities were not reported in those of children with deafblindness. Negative emotions such as anger are commonly experienced by siblings of children with other disabilities (undefined physical or intellectual disabilities) (Seligman, 1983; Hallberg, 2013). These emotions, however, were not reported in siblings of children with deafblindness within our research. Siblings of children with intellectual disabilities may feel threatened by the possibility of death, disability, or contamination (McHale and Gamble, 1989). Also, guilt can inhibit aggressive feelings and lead to depressive symptoms for siblings of children with hemiparesis or physical or mentaldisabilities (Meynckens-Fourez, 1995; Griot et al., 2010; Dufreche Rastello, 2020). Feelings of loneliness, lack of social support, and withdrawal are also common challenges faced by siblings of children with facial paralysis or other undefined physical disabilities (Crocker, 1981; Pogossian, 2003; Guyard, 2012). These issues were not identified in our study. Differences in rules and increased sibling rivalry can lead to aggression and behavioral problems for some siblings of children with facial paralysis or other undefined physical disabilities (Breslau et al., 1981; Crocker, 1981; Lamarche, 1985; Meynckens-Fourez, 1995). Siblings of children with hemiparesis may exhibit behavioral issues to gain attention or wish to be disabled themselves (Dufreche Rastello, 2020). However, behavioral problems for siblings of individuals with deafblindness were not identified.

It is unclear if these aspects are absent, not investigated, or not expressed by the siblings in interviews due to modesty or fear. Also, Smith and Perry (2005) noticed that limited contact with professionals and guilt often prevent siblings of children with a disability from asking questions. Additionally, the desire to appear positive to the interviewer may have led to the concealment of information, such as anger, self- or hetero-aggressive behaviors, social difficulties, and feelings of loneliness. It is also possible that these aspects are less prevalent because the research primarily focused on more well-known syndromes such as Usher and CHARGE, for which more information and support groups are available, potentially introducing a representativeness bias.

For example, in Vert et al. (2016) study, siblings of people with deafblindness reported experiencing somewhat lower levels of loneliness, which contradicts earlier research suggesting higher levels of loneliness among siblings of children with intellectual disabilities (Rossiter and Sharpe, 2001). One possible explanation for this discrepancy is that families with a child with CHARGE syndrome have easy access to support systems through the Internet and various networks. While not many participants actually reported that they attended a CHARGE conference or a support group, the availability of these networks may have helped alleviate any potential feelings of loneliness associated with having a sibling with a disability.

Other factors influencing the results including socioeconomic status, past attendance at a sibling support group, parent stress, family time and routines, family problem-solving and communication, and family hardiness. However, these factors were rarely presented in the selected studies, and thus, we could not examine their detailed impact.

The self-esteem of siblings of children with deafblindness was not explored in our study/ Considering the psychological and social challenges siblings of children with deafblindness are facing, it would be interesting to investigate the consequences on their self-esteem. Previous research suggests that self-esteem in siblings of disabled children (with pervasive developmental disorder) is similar to that of other children (Ferrari et al., 1988). Positive effects such as compassion, empathy, and resilience can develop through the sibling bond (Powell et al., 1985; Metzger, 2005; Scelles, 2008; Griot et al., 2010; Von Benedek, 2013). According to Mchale and Gamble (1989) siblings of children with autism may also acquire responsibility and develop good self-esteem (McHale and Gamble, 1989). Also, for siblings of children with a pervasive developmental disorder or hemiparesis, social behaviors, skills, and creative abilities can be enhanced (Ferrari et al., 1988; Dufreche Rastello, 2020). The lack of results on these variables may be explained by the fact that research initially focused on negative consequences, given the relatively unexplored nature of the field due to the rarity of the disability.

## Limitations

Our research is predicated upon the foundation of preceding studies, which are not without their limitations (e.g., sample size, methodology, and generalizability of results). It is recognized that these limitations can limit our own research. One prominent limitation and bias observed in the antecedent studies is the use of the voluntary participation method. This method has the propensity to introduce bias in the resultant data by attracting individuals with positive attitudes or specific interests, thereby potentially impinging upon the representativeness of the sample and the generalizability of the findings.

Sample size is another limitation— posing challenges in extrapolating the findings to encompass the experience of other siblings of children with deafblindness. It is thus imperative to consider this limitation and its consequential impact when interpreting the findings.

Additionally, relying solely on a single interview with each participant is another significant limitation of the antecedent research and our subsequent use/interpretation of these studies. This approach can influence participant responses, engendering socially desirable answers that may deviate from genuine responses. Consequently, the validity of the results and the comprehension of sibling relationships may be compromised.

Cultural differences can substantially influence the results and impede the transferability of studies concerning the impact of disabilities on families across different cultures. We must recognize and account for these cultural disparities when interpreting and applying the findings.

Furthermore, the inability to access texts not available in English and French presents an additional limitation in our research. This constraint may engender incomplete information and potentially result in overlooking pertinent insights.

It is important to note that specific texts could only be partially accessed in certain cases, with only the title or abstract being available. This further curtails our access to comprehensive information and a holistic understanding of those studies. The absence of full-text access may have omitted valuable details and insights.

The limited number of selected studies and the inability to access texts in other languages underscores the necessity for future research endeavors characterized by enhanced methodologies and augmented sample sizes (that ideally encompass greater diversity). Such endeavors will surmount these limitations and engender more robust, dependable, and generalizable results within the domain of deafblindness research.

## Recommendations

### Practical implications

In light of the challenges some siblings face, we recommend providing psychotherapeutic spaces^2^ to address their needs (Rowan, 1990; Ellis and Hodges, 2013). The research we examined underscores, either explicitly or implicitly, the importance of support and care for siblings in (1) understanding the condition of deafblindness better, (2) expressing their emotions, and (3) acquiring information (Rowan, 1990; Ellis and Hodges, 2013). However, such therapeutic spaces for siblings remain limited, potentially due to a lack of awareness among parents and institutions (Plumridge et al., 2011).

To meet these needs, we recommend employing the Taylor’s Siblings Questionnaire to identify the concerns of siblings of children with deafblindness and tailor interventions accordingly (Rowan, 1990). Professionals like psychologists and psychometricians can facilitate sibling groups and offer a safe environment for siblings to express themselves and develop a deeper understanding of disability-related issues (Pitman and Matthe, 2004; Dufreche Rastello, 2020). Participation in sibling groups has demonstrated numerous benefits, including reduced anxiety, enhanced self-esteem, and improved communication within the family (Heiney et al., 1990; Smith and Perry, 2005; Scelles et al., 2007; Plumridge et al., 2011; Scelles, 2011).

Professionals working with families should acknowledge the impact of disability on both siblings and parents, adopting a comprehensive approach to address their needs (Vert et al., 2016). Support programs should actively involve siblings in crucial educational and transitional meetings and provide sibling workshops to equip them with knowledge and skills (Laman, 2006).

### Research implications

Future studies should incorporate diverse data sources to ensure reliable data collection (Vert et al., 2016). Further exploration of the role of interveners in supporting siblings and the implementation of larger-scale studies will contribute to a deeper understanding of their needs (Vert et al., 2016).

In the context of deafblindness, long-term studies focusing on interventions targeting psychological well-being, orientation and mobility, independence development, transition to adulthood, and sibling relationships are warranted (Rowan, 1990). Additional research is needed to delve into the positive aspects, coping resources, and resilience developed by siblings (Rowan, 1990).

To enhance the generalizability of research, it is recommended to include families from diverse geographical regions and incorporate the perspectives of siblings and children with deafblindness (Rowan, 1990). Conducting longitudinal studies on sibling relationships over time would provide valuable insights, particularly for children with deafblindness. This study highlights the importance of addressing limited interaction or avoidance between siblings and their brother/sister with deafblindness by providing tailored information and support for sibling relationships (Rowan, 1990).

Furthermore, future research should focus on larger and more diverse samples, employ improved methodologies, and directly incorporate siblings’ perspectives to ensure more reliable and generalizable results in the field of deafblindness research.

## Author contributions

MA did the mini-review. RP leads the research project of which this mini-review is a contribution and coordinated MA’s work during the selection process and participated in the corrections. ND corrected the text and provided perspectives. All authors contributed to the article and approved the submitted version.

## Footnotes

^1^In this context, siblings refer to each of the two or more children or offspring who share one or both parents, specifically brothers or sisters.

^2^A therapeutic space is a specially designed environment that supports healing and therapy. It can be a physical or virtual space where individuals engage in therapeutic activities to improve their well-being. It promotes safety, comfort, and relaxation, and may include tools and materials to enhance the therapeutic experience.
